# Diabetes outcomes in heart failure patients with hypertrophic cardiomyopathy

**DOI:** 10.3389/fphys.2022.976315

**Published:** 2022-11-11

**Authors:** Menatalla Mekhaimar, Moza Al Mohannadi, Soha Dargham, Jassim Al Suwaidi, Hani Jneid, Charbel Abi Khalil

**Affiliations:** ^1^ Research department, Weill Cornell Medicine-Qatar, Doha, Qatar; ^2^ Department of Medicine, Icahn School of Medicine at Mount Sinai, New York, NY, United States; ^3^ Heart Hospital, Hamad Medical Corporation, Doha, Qatar; ^4^ Department of Internal Medicine, University of Texas Medical Branch (UTMB), Galveston, TX, United States; ^5^ Joan and Sanford I, Weill Department of Medicine, Weill Cornell Medicine, New York, NY, United States

**Keywords:** hypertrophic cardiomyopathy, heart failure, diabetes, cardiovascular disease, NIS database, heart failure, diabetes

## Abstract

**Aims:** We aimed to assess diabetes outcomes in heart failure (HF) patients with hypertrophic cardiomyopathy (HCM).

**Methods:** The National Inpatient Sample database was analyzed to identify records from 2005 to 2015 of patients hospitalized for HF with concomitant HCM. We examined the prevalence of diabetes in those patients, assessed the temporal trend of in-hospital mortality, ventricular fibrillation, atrial fibrillation, and cardiogenic shock and compared diabetes patients to their non-diabetes counterparts.

**Results:** Among patients with HF, 0.26% had HCM, of whom 29.3% had diabetes. Diabetes prevalence increased from 24.8% in 2005 to 32.7% in 2015. The mean age of patients with diabetes decreased from 71 ± 13 to 67.6 ± 14.2 (*p* < 0.01), but the prevalence of cardiovascular risk factors significantly increased. In-hospital mortality decreased from 4.3% to 3.2% between 2005 and 2015. Interestingly, cardiogenic shock, VF, and AF followed an upward trend. Age (OR = 1.04 [1.03–1.05]), female gender (OR = 1.50 [0.72–0.88]), and cardiovascular risk factors were associated with a higher in-hospital mortality risk in diabetes. Compared to non-diabetes patients, the ones with diabetes were younger and had more comorbidities. Unexpectedly, the adjusted risks of in-hospital mortality (aOR = 0.88 [0.76–0.91]), ventricular fibrillation (aOR = 0.79 [0.71–0.88]) and atrial fibrillation (aOR 0.80 [0.76–0.85]) were lower in patients with diabetes, but not cardiogenic shock (aOR 1.01 [0.80–1.27]). However, the length of stay was higher in patients with diabetes, and so were the total charges per stay.

**Conclusion:** In total, we observed a temporal increase in diabetes prevalence among patients with HF and HCM. However, diabetes was paradoxically associated with lower in-hospital mortality and arrhythmias.

## 1 Introduction

Hypertrophic cardiomyopathy (HCM) is a structural disease of the heart; it can be caused by genes affecting the heart muscle, defined by an increase in myocardial wall thickness of >15 mm in adults (or >13 mm in adults with a first-degree relative with HCM) (Seferović et al., 2019). HCM can also be seen as a result of longstanding hypertension and metabolic disease, causing remodeling of the heart (Olivotto et al., 2013). The structural abnormalities lead to many complications, including arrhythmias, angina, outflow tract obstruction, and heart failure (HF). While in these patients, the leading cause of death is arrhythmias causing sudden cardiac death (SCD) (Tripathi et al., 2019), HF exacerbations account for a common presentation in symptomatic patients, usually with dyspnea on exertion being the primary symptom (Wigle et al., 1995).

Macrovascular complications are the leading cause of death in patients with diabetes (Huang et al., 2017) despite the recent temporal decrease in complications-related mortality (Abi Khalil et al., 2012). Diabetes increases the risk of HF in the general population; it is associated with higher long-term mortality in patients with established HF(Lehrke and Marx, 2017; Kenny and Abel, 2019). Further, a new entity called “diabetic cardiomyopathy” has been recently recognized as a separate entity having concentric hypertrophy and diastolic dysfunction as the main hallmarks, even in the absence of coronary artery disease (CAD) (Dillmann, 2019). Diabetes is often encountered in heart failure with preserved ejection fraction (HFpEF) and is associated with a worse outcome (Lehrke and Marx, 2017).

Gven the increasing prevalence and incidence of diabetes (Seferović et al., 2018), it is inevitable that the proportion of patients with cardiac diseases also increases. We, therefore, assessed the temporal changes in diabetes prevalence in patients with HCM and subsequent cardiovascular and socio-economic outcomes.

## 2 Methods

### 2.1 Data source

Data were extracted from the national inpatient sample (NIS) database between 2005 and 2015. The database is the largest all-payer database in the US, representing almost 20% of inpatient hospitalizations in the US, containing de-identified data, and providing confidentiality. Data is coded using the International Classification of Disease (ICD) ninth edition until 2014, then the 10th edition. This study received approval from Weill Cornell Medicine’s IRB (18-00017).

### 2.2 Diagnosis and outcomes

We analyzed admissions for HF (primary diagnosis), aged 18 or more, with previously reported HCM (secondary diagnosis). Patients were further divided into two groups according to diabetes. All diagnoses were based on ICD-9 and 10 (see Supplementary Appendix). The primary outcome was the prevalence of diabetes in all patients with HF and HCM. Secondary outcomes were in-hospital mortality, cardiogenic shock, ventricular fibrillation (VF), and atrial fibrillation (AF), knowing that sudden cardiac death and ventricular arrhythmias are the most serious and lethal complications of HCM (Houston and Stevens, 2014). Secondary outcomes included socio-economic outcomes, which are the total charges/stay and the length of stay (LoS). We first analyzed the baseline characteristics and cardiovascular and socioeconomic trends of all patients with HCM hospitalized for HF. We then stratified them into two groups according to the presence of diabetes. Further, we merged both groups for intercomparison. Finally, we assessed the predictors of outcomes in patients with diabetes, HF, and HCM.

### 2.3 Statistical methods

Data weighting was performed for the results to be more representative of the nationwide population (around 95% after weighting), as recommended by the Healthcare Cost and Utilization Project, the custodian of the NIS database (AHRQ, 2021). Data analysis was performed using the methodological standards in research using the NIS database (Khera et al., 2017). Trend weight was used for weighting data prior to 2012 and discharge weight from data from 2012 to 2015. Variables were presented using means (standard deviations), medians (interquartile ranges), or numbers (percentages) as deemed appropriate. Temporal trends were analyzed using a linear model. Comparisons of diabetic and non-diabetic patients were made using a Student’s t-test or Chi-square test. We also calculated the Elixhauser score, which includes 31 characteristics that are predictors of poor long-term prognosis and higher mortality risk (Elixhauser et al., 1998). Cardiovascular events were adjusted for factors that were different between both groups, which included: age, gender, race, income, primary expected payer, obesity, hypertension, dyslipidemia, peripheral vascular disease (PVD), chronic kidney disease (CKD), and coronary artery disease (CAD). Multivariable logistic regression was used to assess predictors of cardiovascular events. Total charges/stay were adjusted for yearly inflation, relying on US Bureau of Labor Statistics numbers. Analysis was done using SPSS (IBM, version 26).

## 3 Results

### 3.1 Studied population

**FIGURE 1:**
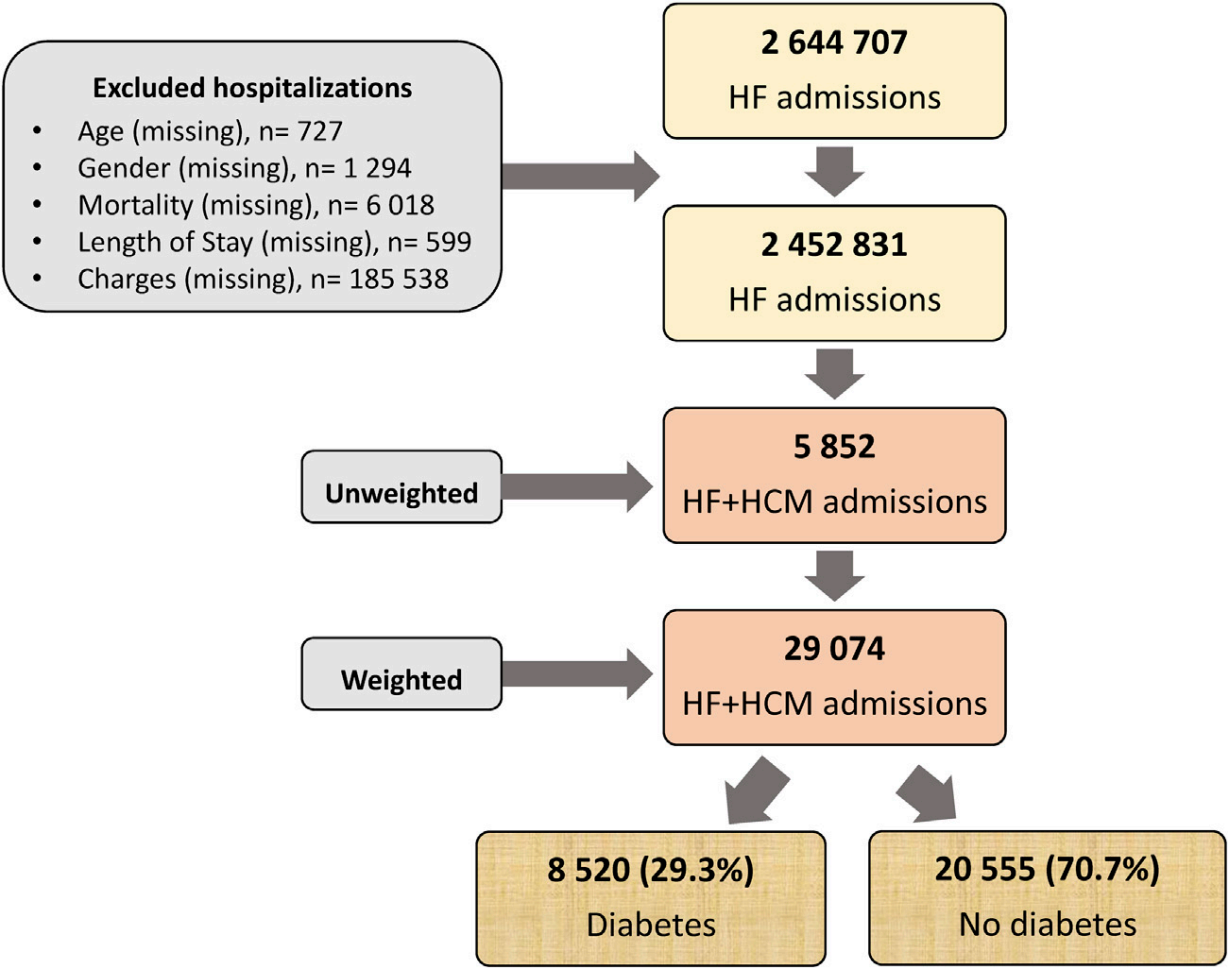
Flow chart of the study. HCM, Hypertrophic cardiomyopathy; HF, heart failure.

We initially included 2 644 707 admissions for HF between 2005 and 2015, of which we assessed 2 452 831 after excluding those with missing records (Figure 1), 5.852 (0.26%) had HCM. Weighted, the number amounted to 29.074 patients being analyzed with HF and HCM. Of those, 8.520 (29.3%) had diabetes and 20.555 (70.7%) did not.

### 3.2 Diabetes prevalence, trends, and outcomes in heart failure patients with hypertrophic cardiomyopathy

First, we looked at the prevalence of diabetes in patients and HF and HCM. As shown in Supplementary Table S1, the prevalence of diabetes increased from 24.8% in 2005 to 33% in 2015 (*p* < 0.001). The same applies to the age-adjusted prevalence, which increased from 22.3% to 32.6%, and the age and sex-adjusted from 22% to 32.9% (*p* < 0.001 for all).

Concomitantly, a temporal increase in the prevalence of hypertension, dyslipidemia, smoking, and other CVD was also noted. In diabetes patients with HCM, the mean (SD) age decreased during the observation period from 71 (13) to 67.6 (14) (*p* = 0.027), with the proportion of those aged <55 increasing over time from 11.2% to 20.9% (*p* < 0.001) (Table 1). There were more females than males. However, the percentage of males increased from 33.2% to 37.8% (*p* = 0.001). White Americans represented up to 75% of the patients in 2005. However, this number decreased to 54.3% in 2015, coupled with an increase in the proportion of Blacks (*p* < 0.001). All cardiovascular risk factors–except hypertension and a history of CAD-increased with time, which was translated to an increase in the Elixhauser score from 1.8 (6) in 2005 to 6.2 (8) in 2015 (*p* < 0.001).

**TABLE 1:** Baseline characteristics and temporal trends of diabetes patients with heart failure and hypertrophic cardiomyopathy, from 2005 to 2015.

Years	2005	2006	2007	2008	2009	2010	2011	2012	2013	2014	2015	P (trend)
Age												
Mean Age (SD)	71.0 (13.0)	68.5 (12.8)	70.7 (14.1)	69.5 (14.0)	69.8 (16.0)	67.7 (11.3)	70.1 (13.8)	67.4 (13.9)	68.1 (14.7)	69.0 (13.8)	67.6 (14.2)	0.027
Age: <55	51 (11.2%)	48 (11.7%)	66 (15.6%)	78 (16.5%)	69 (19.4%)	72 (15.3%)	91 (14.7%)	180 (15.2%)	250 (18.2%)	255 (18.8%)	290 (20.9%)	<0.001
Age: 55–64	69 (15.2%)	90 (21.9%)	43 (10.2%)	92 (19.5%)	63 (17.7%)	111 (23.6%)	98 (15.7%)	255 (21.5%)	240 (17.5%)	180 (13.2%)	275 (19.8%)	<0.001
Age: 65–74	147 (32.4%)	139 (33.8%)	125 (29.6%)	86 (18.2%)	73 (20.6%)	147 (31.3%)	154 (24.6%)	325 (27.4%)	315 (23.0%)	315 (23.2%)	325 (23.4%)	<0.001
Age: 75–84	133 (29.3%)	86 (20.9%)	130 (30.7%)	138 (29.2%)	73 (20.6%)	117 (24.9%)	203 (32.4%)	275 (23.2%)	355 (25.9%)	430 (31.6%)	320 (23.0%)	<0.001
Age: >85	54 (11.9%)	48 (11.7%)	59 (13.9%)	79 (16.7%)	97 (27.3%)	23 (4.9%)	79 (12.6%)	150 (12.7%)	210 (15.3%)	180 (13.2%)	180 (12.9%)	<0.001
Gender												
Male	151 (33.2%)	131 (31.8%)	164 (38.8%)	141 (29.8%)	132 (37.2%)	132 (28.1%)	232 (37.1%)	450 (38.0%)	570 (41.6%)	530 (39.0%)	525 (37.8%)	0.001
Race												
White	272 (74.5%)	201 (60.5%)	201 (60.2%)	282 (72.7%)	170 (61.4%)	248 (55.6%)	339 (59.5%)	630 (57.0%)	805 (61.9%)	785 (60.2%)	720 (54.3%)	<0.001
Black	49 (13.4%)	110 (33.1%)	71 (21.3%)	72 (18.6%)	51 (18.4%)	98 (22.0%)	145 (25.4%)	340 (20.8%)	335 (25.8%)	390 (29.9%)	410 (30.9%)	<0.001
Hispanic	26 (7.1%)	5 (1.5%)	29 (8.7%)	14 (3.6%)	37 (13.4%)	81 (18.2%)	63 (11.1%)	55 (5.0%)	90 (6.9%)	80 (6.1%)	95 (7.2%)	0.001
Asian	13 (3.6%)	0 (0.0%)	16 (4.8%)	5 (1.3%)	11 (4.0%)	19 (4.3%)	14 (2.5%)	15 (1.4%)	25 (1.9%)	10 (0.8%)	40 (3.0%)	0.003
Income												
Low	92 (20.2%)	112 (27.2%)	152 (36.4%)	98 (21.9%)	85 (24.8%)	196 (44.5%)	205 (32.7%)	355 (30.2%)	350 (26.3%)	425 (31.6%)	465 (35.0%)	<0.001
Low-Mid	133 (29.2%)	97 (23.5%)	77 (18.4%)	130 (29.0%)	118 (34.4%)	93 (21.1%)	155 (24.8%)	300 (25.5%)	350 (26.3%)	390 (29.0%)	360 (27.1%)	<0.001
High-Mid	98 (21.5%)	98 (23.8%)	80 (19.1%)	112 (25.0%)	94 (27.4%)	69 (15.7%)	164 (26.2%)	255 (21.7%)	275 (20.7%)	240 (17.8%)	315 (23.7%)	<0.001
High	133 (29.2%)	105 (15.5%)	109 (26.1%)	108 (24.1%)	46 (13.4%)	82 (18.6%)	102 (16.3%)	265 (22.6%)	355 (36.7%)	290 (21.6%)	190 (14.3%)	<0.001
Insurance												
Medicare	332 (72.8%)	295 (71.6%)	322 (76.3%)	338 (72.1%)	233 (65.6%)	297 (62.2%)	478 (76.2%)	780 (66.1%)	930 (67.9%)	1050 (77.2%)	910 (65.5%)	<0.001
Medicaid	43 (9.4%)	44 (10.7%)	22 (5.2%)	24 (5.1%)	46 (13.0%)	92 (19.6%)	59 (9.4%)	130 (11.0%)	115 (8.4%)	90 (6.6%)	205 (14.7%)	<0.001
Private	60 (13.2%)	64 (15.5%)	59 (14.0%)	83 (17.7%)	66 (18.6%)	56 (11.9%)	62 (9.9%)	225 (19.1%)	270 (19.7%)	185 (13.6%)	215 (15.5%)	<0.001
Self-Pay	16 (3.5%)	4 (1.0%)	10 (2.4%)	5 (1.1%)	0 (0.0%)	25 (5.3%)	23 (3.7%)	25 (2.1%)	20 (1.5%)	25 (1.8%)	40 (2.9%)	0.001
Comorbidities												
Obesity	85 (18.7%)	81 (19.7%)	79 (18.7%)	415 (12.4%)	80 (22.5%)	137 (29.1%)	158 (25.3%)	395 (33.3%)	430 (31.4%)	475 (34.9%)	500 (36.0%)	<0.001
Hypertension	292 (64.2%)	245 (59.5%)	282 (66.8%)	302 (63.8%)	249 (70.1%)	350 (74.3%)	426 (68.1%)	835 (70.5%)	940 (68.8%)	945 (69.5%)	1060 (76.3%)	0.085
Smoking	47 (10.3%)	63 (5.3%)	9 (2.1%)	53 (11.2%)	49 (13.8%)	112 (23.8%)	149 (23.8%)	345 (29.1%)	355 (25.9%)	395 (29.0%)	455 (32.7%)	<0.001
Dyslipidemia	142 (31.1%)	114 (27.7%)	182 (43.1%)	157 (33.1%)	176 (49.6%)	242 (51.4%)	338 (54.0%)	655 (55.3%)	765 (55.8%)	790 (58.1%)	855 (61.5%)	<0.001
Past Medical History												
PVD	33 (7.2%)	19 (4.6%)	37 (8.7%)	50 (10.6%)	44 (12.4%)	45 (9.6%)	73 (11.7%)	100 (8.4%)	160 (11.7%)	165 (12.1%)	170 (12.2%)	<0.001
CKD	59 (13.0%)	85 (20.6%)	111 (26.2%)	192 (40.5%)	100 (28.2%)	145 (30.8%)	278 (44.5%)	580 (48.9%)	600 (43.8%)	650 (47.8%)	725 (52.2%)	<0.001
CAD	193 (42.3%)	136 (33.0%)	114 (27.0%)	166 (35.0%)	163 (46.0%)	171 (36.3%)	258 (41.2%)	525 (44.3%)	560 (40.9%)	575 (42.3%)	525 (37.8%)	0.309
Elixhauser score	1.8764 (6.78)	1.7439 (7.02)	1.4576 (6.89)	4.2394 (7.86)	4.4835 (8.93)	4.4835 (8.93)	4.4692 (7.09)	4.5150 (8.82)	4.6765 (8.26)	5.2900 (7.96)	6.2188 (8.64)	0.001
Mean (SD)												
Outcomes												
Cardiogenic shock	11* (0.6%)	12 (0.3%)	11* (0.7%)	11* (1.0%)	11* (0.4%)	11* (1.9%)	11 (1.8%)	25 (2.1%)	30 (2.2%)	20 (1.5%)	30 (2.2%)	<0.001
Ventricular fibrillation	11* (2.2%)	43 (10.4%)	15 (3.5%)	9 (1.9%)	24 (6.8%)	34 (7.2%)	38 (6.1%)	105 (8.9%)	95 (6.9%)	110 (8.1%)	110 (7.9%)	<0.001
Atrial fibrillation	175 (38.5%)	142 (34.5%)	130 (30.7%)	209 (44.1%)	122 (34.4%)	144 (30.6%)	294 (47.0%)	485 (40.9%)	645 (47.1%)	715 (52.6%)	660 (47.5%)	<0.001

CAD , coronary artery disease; CKD , chronic kidney disease; PVD , Peripheral vascular disease. *Per the requirements of the HCUP, cells less or equal to 10 are noted as < 11.

**FIGURE 2:**
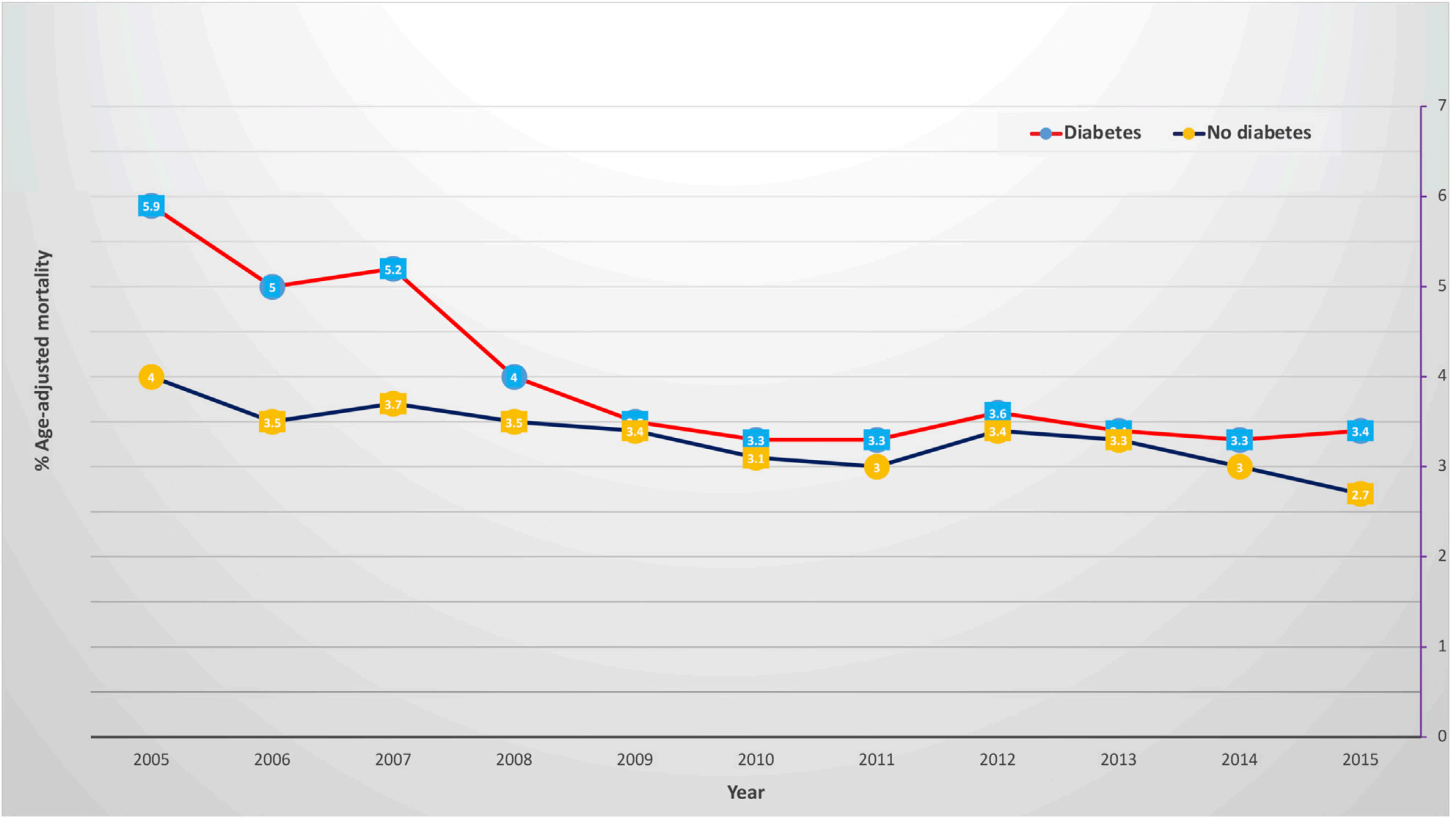
In-hospital mortality trend in patients with hypertrophic cardiomyopathy, heart failure, and diabetes (red color) and without diabetes (blue color). The X-axis represents the percentage of in-hospital mortality. The Y-axis represents the year.

When looking at the temporal trends of outcomes, we found that crude in-hospital mortality decreased from 4.3% to 3.2% (<0.001) in patients with diabetes (Figure 2). Interestingly, cardiogenic shock, VT, and AF followed an upward trend (*p* < 0.001 for all). Similar temporal trends exist regarding decreasing age, gender distribution, and rising prevalence of obesity, smoking, and dyslipidemia in patients without diabetes (Supplementary Table S2). In-hospital crude mortality also significantly decreased during the observation period in non-diabetes patients (3.9% in 2005 vs 2.7% in 2015, *p* = 0.009) (Figure 2).

### 3.3 Comparing diabetes to non-diabetes patients

After merging all years, we first compared the baseline characteristics of diabetes to non-diabetes patients. Most patients are within the 75–84 age group in both populations. Non-diabetes patients tended to be older, with 24.1% of them aged >85 compared to 13.1% in the diabetes group (*p* < 0.001) (Table 2). There were more females than males in all patients; however, a higher percentage of females is observed in the non-diabetes group (66% vs 62.9%, *p* < 0.001). As expected, a significantly higher number of patients with diabetes were obese and had hypertension and dyslipidemia. Additionally, PVD and CKD were more prevalent in the diabetes group. Unexpectedly, diabetes patients hospitalized for HF with HCM had significantly lower in-hospital mortality (3.9%) compared to non-diabetic ones (3.3%) (*p* < 0.001). After adjustments on characteristics that were different among both groups (Table 3), diabetes was associated with an adjusted lower in-hospital mortality rate (aOR = 0.84 [0.74–0.96]) (Table 4). The adjusted risk of cardiogenic shock was similar in both groups (aOR = 1.01 [0.80–1.27]. However, patients with diabetes also had a lower adjusted risk of ventricular fibrillation (aOR 0.79 CI [0.71–0.88]) and atrial fibrillation (aOR 0.8 CI [0.76–0.85]).

**TABLE 2:** Baseline characteristics of patients with heart failure and hypertrophic cardiomyopathy, with and without diabetes.

	Non-diabetes N = 20,555	Diabetes N = 8,520	*p* value
Age			
Mean (SD)	70.44 (16.66)	68.99 (13.97)	<0.001
<55	3916 (19.1%)	1453 (17.1%)	<0.001
55–64	2945 (14.3%)	1516 (17.8%)	<0.001
65–74	3395 (16.5%)	2150 (25.2%)	<0.001
75–84	5345 (26.0%)	2285 (26.8%)	<0.001
>85	4954 (24.1%)	1116 (13.1%)	<0.001
Gender			
Male	6994 (34.0%)	3158 (37.1%)	<0.001
Female	13560 (66.0%)	5362 (62.9%)	<0.001
Race			
White	13404 (73.8%)	4652 (60.1%)	<0.001
Black	3115 (17.2%)	2070 (26.7%)	<0.001
Hispanic	788 (4.3%)	574 (7.4%)	<0.001
Asian	337 (1.9%)	168 (2.2%)	<0.001
Income			
Low	5116 (25.5%)	2535 (30.5%)	<0.001
Low-Mid	5192 (25.8%)	2204 (26.5%)	<0.001
High-Mid	4780 (23.8%)	1799 (21.6%)	<0.001
High	5011 (24.9%)	1784 (21.4%)	<0.001
Insurance			
Medicare	14329 (69.8%)	5965 (70.1%)	<0.001
Medicaid	1508 (7.3%)	870 (10.2%)	<0.001
Private Insurance	3794 (18.5%)	1346 (25.8%)	<0.001
Self-Pay	587 (2.9%)	192 (2.3%)	0.004
Comorbidities			
Obesity	2831 (13.8%)	2480 (29.1%)	<0.001
Hypertension	12006 (58.4%)	5927 (69.6%)	<0.001
Smoking	5059 (24.6%)	2033 (23.9%)	0.171
Dyslipidemia	7360 (35.8%)	4416 (51.8%)	<0.001
Past Medical History			
PVD	1628 (7.9%)	897 (10.5%)	<0.001
CKD	5553 (27.0%)	3525 (41.4%)	<0.001
CAD	6243 (30.4%)	3385 (39.7%)	<0.001
Elixhauser score	4.53 (8.07)	5.48 (8.29)	<0.001

CAD , coronary artery disease; CKD , chronic kidney disease; PVD , peripheral vascular disease.

**TABLE 3:** Multivariable regression of in-hospital mortality among diabetes patients with heart failure and hypertrophic cardiomyopathy.

	Or (95% CI)	*p*-value
Age		
<55	Ref	Ref
55–64	1.21 (0.87–1.67)	0.258
65–74	1.45 (1.04–2.01)	0.028
75–84	1.59 (1.23–2.34)	0.001
>85	2.93 (2.13–4.05)	<0.001
Mean	1.66 (1.44–1.99)	<0.001
Gender		
Male	Ref	Ref
Female	1.38 (1.16–1.64)	<0.001
Race		
White	Ref	Ref
Black	0.97 (0.78–1.21)	0.812
Hispanic	0.93 (0.64–1.34)	0.678
Asian	0.926 (0.53–1.61)	0.784
Income		
Low	Ref	Ref
Low-Mid	1.02 (0.82–1.26)	0.874
High-Mid	1.03 (0.82–1.27)	0.827
High	109 (0.88–1.36)	0.437
Insurance		
Medicare	Ref	Ref
Medicaid	0.46 (0.28–0.75)	0.002
Private Insurance	0.91 (0.70–1.18)	0.479
Self-Pay	1.47 (0.89–2.42)	0.135
Comorbidities		
Obesity	1.85 (1.48–2.30)	<0.001
Hypertension	0.79 (0.68–0.92)	0.002
Smoking	Not in model	-
Dyslipidemia	0.73 (0.62–0.86)	<0.001
Past Medical History		
PVD	1.32 (1.07–1.62)	0.01
CKD	0.88 (0.75–1.04)	0.138
CAD	0.79 (0.67–1.03)	0.051
Elixhauser score	1.10 (1.09–1.11)	<0.001

CAD , coronary artery disease; CKD , chronic kidney disease; PVD , peripheral vascular disease.

**TABLE 4:** Cardiovascular outcomes of patients with heart failure and hypertrophic cardiomyopathy, with and without diabetes.

	Non-diabetes	Diabetes	Adjusted OR (95% CI)
In-hospital mortality, n (%)	678 (3.3%)	332 (3.9%)	0.88 (0.76–0.91)
OR (95% CI)	1	0.84 (0.74–0.96)	
Ventricular fibrillation n (%)	1745 (8.5%)	594 (7.0%)	0.79 (0.71–0.88)
OR (95% CI)	1	0.81 (0.73–0.89)	
Atrial fibrillation, n (%)	10028 (48.8%)	3636 (42.7%)	0.80 (0.76–0.85)
OR (95% CI)	1	0.79 (0.74–0.83)	
Cardiogenic shock, n (%)	442 (2.2%)	151 (1.9%)	1.01 (0.80–1.27)
OR (95% CI)	1	0.84 (0.72–0.89)	

### 3.4 Socioeconomic outcomes

**FIGURE 3:**
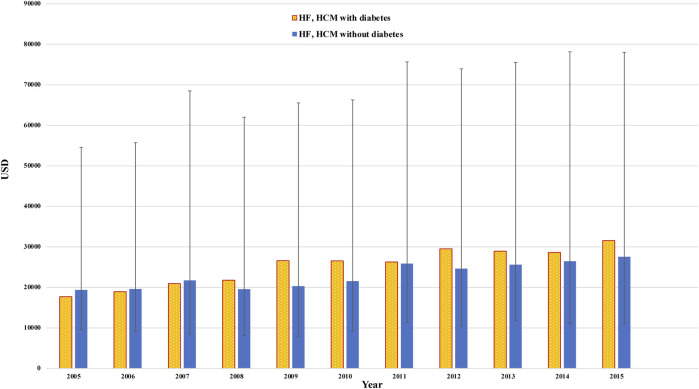
Temporal change in total charges/stay (median +/− IQR) in heart failure patients with diabetes (yellow color) and without diabetes (blue color). The X-axis represents the amount in USD. The Y-axis represents the year.

In diabetes and non-diabetes patients, hospitalization charges significantly increased between 2005 and 2015. The cost of hospitalizations in diabetes patients was lower up to 2007, but by 2008, diabetes patients had significantly higher charges, which continued until 2015 (Figure 3). We did not observe a temporal change in the length of stay (LoS). Further, patients with diabetes had a higher median (IQR) length of stay (5 [3–7] vs 4 [3–6] days, diabetes *versus* non-diabetes, *p* < 0.001).

### 3.5 D- predictors of outcomes in patients with diabetes

In-hospital mortality increased by almost 8-fold in those aged >85 years old (OR 7.49 [2.88–19.45], *p* < 0.001) and in females compared to males (OR 1.50 [1.08–2.08], *p* = 0.016). As expected, it also increased with concomitant renal failure (OR 1.35 [1.004–1.82], *p* = 0.047), CAD (OR 1.54 [1.14–2.07], *p* = 0.004), and those with PVD (OR 2.98 [2.11–4.21], *p* < 0.001) (Supplementary Table S3). Consequently, the Elixhauser score increased the in-hospital mortality risk (OR 1.09 [1.07–1.11], *p* < 0.001).

Increasing age did not predict cardiogenic shock (Supplementary Table S4), but the female gender was protective (OR 0.47 [0.31–0.71], *p* < 0.001). Asians and Native Americans had a higher risk compared to Whites. Obesity was also associated with higher rates of cardiogenic shock (OR 2.27 [1.42–3.65], *p* < 0.001), and so was having CAD (OR 2.41 [1.57–2.30], *p* < 0.001) and renal failure (OR 1.31 [1.19–1.50), *p* < 0.001).

Patients aged 55 to 74 had a higher risk of developing ventricular fibrillation (Supplementary Table S5) than those younger than 55. However, the female gender was associated with 37% less risk. Age was also associated with a higher risk of atrial fibrillation (OR 1.84 [0.63–0.71] and women had a lower risk (OR 0.67 [0.63–0.71] (Supplementary Table S5). Among cardiovascular risk factors, obesity was the strongest predictor of developing AF (OR 1.42 [1.12–1.95), *p* < 0.001).

## 4 Discussion

We report in this study that the incidence of diabetes is gradually increasing in patients with heart failure and hypertrophic cardiomyopathy. Furthermore, diabetes was surprisingly associated with a lower risk of in-hospital mortality and arrhythmias. However, the length of stay and total charges/stay were higher in patients with diabetes.

Diabetes has deleterious effects on cardiac function. Although the exact mechanism is unclear, there is damage to cardiac cells, and initially, patients have diastolic dysfunction, which eventually progresses to systolic dysfunction (Kenny and Abel, 2019). Diabetic cardiomyopathy is one of the types of non-ischemic cardiomyopathies, and commonly cardiovascular mortality is a cause of progressive HF(Bertoni et al., 2003). This study reports an increase in diabetes prevalence among HF patients with HCM, which corresponds to the rising prevalence of diabetes in the general population and other cardiovascular disorders (Collaboration, 2016). Conrad et al. reported an average 8% increase in diabetes prevalence over 10 years in a cohort of 4 million HF patients in the United Kingdom (Conrad et al., 2018). We recently reported that diabetes prevalence in patients with HF is also increasing in the US(Mekhaimar et al., 2021). Further, diabetes patients in our study are older and have more CVD risk factors, which is also concordant with other studies (Wasserstrum et al., 2019).

Despite the increasing prevalence and the worsening of the cardio-metabolic profile in patients with diabetes, we observed a significant decline in in-hospital mortality, which has been reported in myocardial infarction (Ahmed et al., 2014; Ali et al., 2022), stroke (Tabbalat et al., 2021), HF (Mekhaimar et al., 2021), and valvular heart disease (Khan et al., 2022). In our study, in-hospital mortality in all patients decreased over time, similar to other reported trends for patients with HCM (Elliott et al., 2006; Maron et al., 2015). This could be due to more contemporary procedures specific to HCM, including myomectomies and earlier ICD placement in primary prevention (Maron et al., 2016).

To our knowledge, we are the first to assess the cardiovascular impact effect of diabetes on HF patients with HCM. Wasserstrum et al. showed that concurrent HCM and diabetes, in the absence of HF, lead to worse outcomes, including mortality (Wasserstrum et al., 2019). However, the difference in mortality was only significant in the last 5 years of the 15-year follow-up; the first 5 years of the study showed lower mortality in the presence of diabetes, which is concordant with our results. Another study looking at outcomes after septal myectomy in 201 HCM patients and comparing diabetes *versus* non-diabetes patients found identical mortality in both groups (Wang et al., 2020). Analysis of the OPTIMIZE-HF registry found that diabetes did not affect in-hospital mortality (Greenberg et al., 2007). We recently reported a lower in-hospital mortality risk in diabetes patients with HF compared to non-diabetes in the NIS database (Mekhaimar et al., 2021). It might be possible that the lower in-hospital mortality observed in HF patients with diabetes also applies to patients with HF and HCM.

It is not clear why diabetes was associated with lower in-hospital mortality risk. It might be possible that diabetes patients are closely monitored and better taken care of, including earlier initiation of guideline-directed medical therapy and implantable cardioverter defibrillators (ICD) placement, which would mitigate sudden cardiac death and fatal arrhythmias (Maron et al., 2003; Ranka et al., 2019; Ommen et al., 2020). This would also explain the higher charges and longer LOS we observed. Patients with HCM but without diabetes may appear compensated for longer and more rarely progress to end-stages of heart failure; therefore, they may be less likely to be getting care actively (Maron et al., 2000).

Limitations of this study include the retrospective nature of our data; therefore, we cannot make any conclusions about causality. Further, the NIS is an administrative database that was initially designed to produce national estimates of inpatient utilization, access, cost, quality, and outcomes; hence, there could be potential errors in its utilization in clinical investigations, such as–but not limited to - the accuracy or the lack of accuracy of ICD codes. Furthermore, our data set cannot tell the severity of heart failure, diabetes parameters (type, HBA_1c,_ and duration), and HCM parameters (medications, septal thickness, left ventricular ejection fraction, and the etiology). This information would have allowed us to delineate the relationship between the two disease entities and make further conclusions about why the results came out.

**Table:** Coding of the diagnosis

Diagnosis	ICD9 Codes	ICD10 Codes
Hypertrophic cardiomyopathy	425.1, 425.11, 425.18	I42.1, I42.2
Heart failure	402.01, 402.11, 402.91, 404.01, 404.03, 404.11, 404.13, 404.91, 404.93, and all 428 sub-groups (428.30 to 33)	I11.0, I13.0, I13.2, I50.814, I50.9, I150.1, I150.20, (I150.21 to 23), (I150.40 to 43), (I150.810 to 813), (I150.82 to 84), I1590.89, I50.9, I50.30+I50.31+I50.32+I50.33
Ventricular fibrillation	427.1	I47.2
Atrial fibrillation	427.31	I48.91
Cardiogenic shock	785.51	R57.0

## 5 Conclusion

In this analysis of the National Inpatient Sample database, we report an increase in the prevalence of diabetes in heart failure patients with concomitant hypertrophic cardiomyopathy. The in-hospital mortality in those patients is on a descending slope despite the temporal increase in cardiovascular risk factors. Diabetes was paradoxically associated with a lower in-hospital mortality rate which might be due to early aggressive treatment of those patients as reflected in higher charges and longer lengths of stay compared to their non-diabetic counterparts. Our results must be validated in different populations and, most importantly, in cardiovascular cohorts.

## Data Availability

The raw data supporting the conclusions of this article will be made available by the authors, without undue reservation.
