# Supplementary material for: Diabetes outcomes in heart failure patients with hypertrophic cardiomyopathy
Source: Front Physiol. 2022 Nov 11;13:976315. doi: 10.3389/fphys.2022.976315 (PMC9691891; doi:10.3389/fphys.2022.976315)
Supplement: Supplementary file 1 [file DataSheet1.docx]

**Supplementary Table 1:** Baseline characteristics and temporal trends of HF and HCM from 2005 to 2015.

| **Years** | **2005** | **2006** | **2007** | **2008** | **2009** | **2010** | **2011** | **2012** | **2013** | **2014** | **2015** | **P (trend)** |
| --- | --- | --- | --- | --- | --- | --- | --- | --- | --- | --- | --- | --- |
| **Diabetes** |  |  |  |  |  |  |  |  |  |  |  |  |
| Prevalence | 455 (24.8%) | 412 (21.4%) | 423  (27.0%) | 474 (28.3%) | 355 (20.9%) | 470 (25.3%) | 626 (27.9%) | 1185 (32.3%) | 1370 (32.4%) | 1360 (33.0%) | 1390 (32.7%) | <0.001 |
| Prevalence  (Age adjusted) | 22.3% | 19.1% | 29.2% | 28.4% | 22.8% | 25% | 291% | 31.3% | 32.2% | 30.3% | 32.6% | <0.001 |
| Prevalence  (Age and sex adjusted) | 22% | 19.1% | 29.8% | 29.1% | 22.45% | 24.6% | 23.7% | 31.1% | 32.5% | 32.6% | 32.9% | <0.001 |
| **Age** |  |  |  |  |  |  |  |  |  |  |  |  |
| Mean (SD) | 72.3 (14.9) | 70.5 (15.4) | 72.7 (15.0) | 71.1 (16.7) | 70.4 (16.1) | 69.9 (15.8) | 70.6 (15.8) | 69.6 (16.0) | 69.8 (15.9) | 69.15 (16.2) | 68.4 (16.1) | 0.001 |
| Age: <55 (age) | 248 (13.5%) | 315 (16.4%) | 222 (14.2%) | 318 (19.0%) | 292 (17.2%) | 329 (17.7%) | 445 (19.8%) | 660 (18.0%) | 745 (17.6%) | 855 (20.7%) | 940 (22.1%) | <0.001 |
| Age: 55-64 | 249 (13.6%) | 296 (15.4%) | 161 (10.3%) | 225 (13.4%) | 258 (15.2%) | 399 (21.5%) | 332 (14.8%) | 585 (15.9%) | 695 (16.4%) | 570 (13.8%) | 690 (16.2%) | <0.001 |
| Age: 65 - 74 | 404 (22.1%) | 380 (19.8%) | 272 (17.3%) | 257 (15.4%) | 327 (19.3%) | 312 (16.8%) | 364 (16.2%) | 770 (21.0%) | 810 (19.1%) | 845 (20.5%) | 805 (18.9%) | <0.001 |
| Age: 75-84 | 541 (29.5%) | 568 (29.6%) | 588 (37.5%) | 462 (27.6%) | 458 (27.0%) | 434 (23.3%) | 598 (26.7%) | 870 (23.7%) | 1080 (25.5%) | 1050 (25.5%) | 980 (23.0%) | <0.001 |
| Age: >85 | 390 (21.3%) | 363 (18.9%) | 325 (20.7%) | 412 (24.6%) | 360 (21.2%) | 386 (20.8%) | 503 (22.4%) | 785 (21.4%) | 900 (21.3%) | 805 (19.5%) | 840 (19.7%) | <0.001 |
| **Gender** |  |  |  |  |  |  |  |  |  |  |  |  |
| Male | 581 (31.7%) | 549 (28.5%) | 438 (27.9%) | 415 (24.8%) | 490 (28.9%) | 531 (28.5%) | 789 (35.2%) | 1435 (29.1%) | 1650 (39.0%) | 1596 (38.7%) | 1680 (39.5%) | <0.001 |
| **Race** |  |  |  |  |  |  |  |  |  |  |  |  |
| White | 1081 (79.9%) | 1048 (75.7%) | 829 (67.5%) | 1025 (76.8%) | 970 (73.6%) | 1253 (72.3%) | 1509 (71.6%) | 2395 (68.8%) | 2760 (68.7%) | 2580 (66.5%) | 2605 (64.2%) | <0.001 |
| Black | 151 (11.2%) | 216 (15.6%) | 217 (17.7%) | 209 (15.7%) | 181 (13.7%) | 268 (15.5%) | 398 (18.9%) | 725 (20.8%) | 835 (20.8%) | 960 (24.7%) | 1025 (25.2%) | <0.001 |
| Hispanic | 63 (4.7%) | 51 (3.7%) | 89 (7.2%) | 44 (3.3%) | 82 (6.2%) | 123 (7.1%) | 110 (5.2%) | 190 (5.5%) | 225 (5.6%) | 180 (4.6%) | 205 (5.0%) | <0.001 |
| Asian | 39 (2.9%) | 24 (1.7%) | 55 (4.5%) | 13 (1.0%) | 21 (1.6%) | 44 (2.5%) | 39 (1.9%) | 45 (1.3%) | 70 (1.7%) | 65 (1.7%) | 90 (2.2%) | <0.001 |
| **Income** |  |  |  |  |  |  |  |  |  |  |  |  |
| Low | 399 (22.0%) | 371 (19.8%) | 462 (30.4%) | 393 (24.4%) | 341 (20.5%) | 618 (34.3%) | 608 (27.5%) | 995 (27.7%) | 1085 (26.2%) | 1155 (28.4%) | 1225 (29.7%) | <0.001 |
| Low-Mid | 463 (25.5%) | 548 (29.2%) | 338 (22.2%) | 409 (25.4%) | 466 (28.0%) | 419 (23.3%) | 522 (23.6%) | 980 (27.3%) | 1080 (26.1%) | 1095 (26.9%) | 1075 (26.0%) | <0.001 |
| High-Mid | 446 (24.6%) | 504 (26.9%) | 343 (22.6%) | 371 (23.0%) | 416 (25.0%) | 389 (616%) | 616 (27.8%) | 750 (20.9%) | 950 (23.0%) | 890 (21.9%) | 905 (21.9%) | <0.001 |
| High-Mid | 206 (27.9%) | 451 (24.1%) | 378 (24.9%) | 440 (27.3%) | 439 (26.4%) | 374 (20.8%) | 468 (21.1%) | 865 (24.1%) | 1020 (24.7%) | 930 (22.9%) | 925 (22.4%) | 0.471 |
| **Insurance** |  |  |  |  |  |  |  |  |  |  |  |  |
| Medicare | 1293 (70.6%) | 1354 (70.6%) | 1155 (73.7%) | 1200 (71.9%) | 1220 (71.9%) | 1213 (65.2%) | 1568 (70.0%) | 2575 (70.3%) | 2965 (70.1%) | 2880 (69.9%) | 2870 (67.5%) | <0.001 |
| Medicaid | 142 (7.8%) | 112 (5.8%) | 58 (3.7%) | 91 (5.5%) | 119 (7.0%) | 268 (14.4%) | 198 (8.8%) | 290 (7.9%) | 330 (7.8%) | 310 (7.5%) | 460 (10.8%) | <0.001 |
| Private | 340 (18.6%) | 392 (20.4%) | 280 (17.9%) | 315 (18.9%) | 284 (16.7%) | 302 (16.2%) | 383 (17.1%) | 650 (17.7%) | 750 (17.7%) | 750 (18.2%) | 695 (16.4%) | <0.001 |
| Self-Pay | 49 (2.7%) | 36 (1.9%) | 51 (3.3%) | 28 (1.7%) | 44 (2.6%) | 57 (3.1%) | 74 (3.3%) | 80 (2.2%) | 100 (2.4%) | 110 (2.7%) | 150 (3.5%) | <0.001 |
| **Comorbidities** |  |  |  |  |  |  |  |  |  |  |  |  |
| Obesity | 189 (10.3%) | 154 (8.0%) | 195 (12.4%) | 171 (20.2%) | 189 (11.2%) | 306 (16.4%) | 422 (18.8%) | 735 (20.0%) | 845 (20.0%) | 975 (23.6%) | 1130 (26.6%) | <0.001 |
| Hypertension | 941 (51.4%) | 849 (46.5%) | 870 (55.5%) | 930 (55.6%) | 1047 (61.8%) | 1113 (59.8%) | 1429 (63.7%) | 2305 (62.8%) | 2685 (63.5%) | 2700 (65.5%) | 3020 (71.0%) | 0.126 |
| Smoking | 277 (15.1%) | 223 (11.6%) | 206 (13.1%) | 237 (14.2%) | 291 (17.2%) | 396 (21.3%) | 597 (26.6%) | 955 (26.0%) | 1155 (27.3%) | 1290 (31.3%) | 1465 (34.4%) | <0.001 |
| Dyslipidemia | 434 (23.7%) | 458 (23.8%) | 420 (26.8%) | 529 (31.6%) | 611 (36.0%) | 664 (35.7%) | 950 (42.4%) | 1515 (41.3%) | 2005 (47.4%) | 2020 (49.0%) | 2170 (51.0%) | <0.001 |
| **Past Medical History** |  |  |  |  |  |  |  |  |  |  |  |  |
| PVD | 96 (5.2%) | 130 (6.8%) | 112 (7.1%) | 151 (9.0%) | 107 (6.3%) | 166 (8.9%) | 187 (8.3%) | 295 (8.0%) | 420 (9.9%) | 445 (10.8%) | 415 (9.8%) | <0.001 |
| CKD | 196 (10.7%) | 341 (17.7%) | 341 (21.7%) | 461 (27.5%) | 399 (23.5%) | 481 (25.8%) | 739 (33.0%) | 1380 (37.6%) | 1515 (35.8%) | 1525 (37.0%) | 1700 (40.0%) | <0.001 |
| CAD | 556 (30.3%) | 501 (26.1%) | 451 (28.7%) | 417 (24.9%) | 591 (34.9%) | 579 (31.1%) | 754 (33.6%) | 1400 (38.1%) | 1590 (37.6%) | 1455 (35.3%) | 1335 (31.4%) | 0.354 |
| **Elixhauser score**  Mean (SD) | 3.3866 (6.66) | 3.6406 (7.05) | 4.4659 (7.25) | 4.6409 (7.04) | 5.2537 (8.04) | 5.1257 (8.04) | 5.6944 (8.19) | 5.4174 (8.64) | 5.6191 (8.91) | 5.6242 (8.70) | 6.0382 (8.64) | 0.006 |

*CAD= coronary artery disease. CKD=chronic kidney disease. PVD= Peripheral vascular disease.*

**Supplementary Table 2:** Baseline characteristics and temporal trends of HF and HCM without diabetes from 2005 to 2015

| **Years** | **2005** | **2006** | **2007** | **2008** | **2009** | **2010** | **2011** | **2012** | **2013** | **2014** | **2015** | **P (trend)** |
| --- | --- | --- | --- | --- | --- | --- | --- | --- | --- | --- | --- | --- |
| **Age** |  |  |  |  |  |  |  |  |  |  |  |  |
| Mean (SD) | 72.8 (15.5) | 71.1 (16.0) | 73.4 (15.3) | 71.3 (17.7) | 70.1 (16.1) | 70.6 (16.9) | 70.7 (16.5) | 70.2 (16.8) | 70.3 (16.4) | 68.9 (17.3) | 68.8 (16.9) | 0.005 |
| Age: <55 (age) | 197 (14.3%) | 266 (17.6%) | 156 (13.6%) | 240 (20.0%) | 223 (16.6%) | 257 (18.5%) | 352 (21.8%) | 480 (19.3%) | 495 (17.3%) | 600 (21.7%) | 650 (22.7%) | <0.001 |
| Age: 55-64 | 181 (13.1%) | 205 (13.6%) | 119 (10.4%) | 133 (11.1%) | 195 (14.6%) | 288 (20.7%) | 234 (14.5%) | 330 (13.3%) | 455 (15.9%) | 390 (14.1%) | 415 (14.5%) | <0.001 |
| Age: 65 - 74 | 256 (18.6%) | 242 (16.0%) | 147 (12.8%) | 170 (14.2%) | 253 (28.9%) | 165 (11.9%) | 210 (13.0%) | 445 (17.9%) | 495 (17.3%) | 530 (19.2%) | 480 (16.8%) | <0.001 |
| Age: 75-84 | 408 (29.6%) | 482 (31.9%) | 458 (39.9%) | 324 (27.0%) | 362 (27.0%) | 317 (22.8%) | 395 (24.5%) | 595 (23.9%) | 725 (25.3%) | 620 (22.4%) | 660 (23.0%) | <0.001 |
| Age: >85 | 335 (24.3%) | 315 (20.9%) | 267 (23.3%) | 333 (27.8%) | 307 (22.9%) | 363 (26.1%) | 424 (26.3%) | 635 (25.6%) | 690 (24.1%) | 625 (22.6%) | 660 (23.0%) | <0.001 |
| **Gender** |  |  |  |  |  |  |  |  |  |  |  |  |
| Male | 430 (31.2%) | 418 (27.7%) | 275 (24.0%) | 274 (22.8%) | 358 (26.7%) | 398 (28.6%) | 557 (34.5%) | 985 (39.6%) | 1080 (37.8%) | 1065 (38.5%) | 1155 (40.3%) | <0.001 |
| **Race** |  |  |  |  |  |  |  |  |  |  |  |  |
| White | 810 (82.0%) | 847 (80.4%) | 628 (70.2%) | 744 (78.3%) | 800 (76.8%) | 1005 (78.0%) | 1170 (76.1%) | 1765 (74.3%) | 1955 (71.9%) | 1795 (69.7%) | 1885 (68.9%) | <0.001 |
| Black | 102 (10.3%) | 106 (10.1%) | 146 (16.3%) | 137 (14.4%) | 130 (12.5%) | 170 (13.2%) | 253 (16.5%) | 385 (16.2%) | 500 (18.4%) | 570 (22.1%) | 615 (22.5%) | <0.001 |
| Hispanic | 37 (3.7%) | 46 (4.4%) | 61 (6.8%) | 30 (3.2%) | 46 (4.4%) | 41 (3.2%) | 47 (3.1%) | 135 (5.7%) | 135 (5.0%) | 100 (3.9%) | 110 (4.0%) | <0.001 |
| Asian | 25 (2.5%) | 24 (2.3%) | 38 (4.2%) | 9 (0.9%) | 10 (1.0%) | 26 (2.0%) | 25 (1.6%) | 30 (1.3%) | 45 (1.7%) | 55 (2.1%) | 50 (1.8%) | 0.002 |
| **Income** |  |  |  |  |  |  |  |  |  |  |  |  |
| Low | 308 (22.7%) | 260 (17.8%) | 310 (28.1%) | 249 (25.3%) | 256 (19.4%) | 422 (31.0%) | 403 (24.4%) | 640 (26.5%) | 735 (26.2%) | 730 (26.8%) | 760 (27.1%) | <0.001 |
| Low-Mid | 330 (24.3%) | 451 (30.8%) | 261 (23.6%) | 279 (24.0%) | 348 (26.4%) | 326 (24.0%) | 367 (23.1%) | 680 (28.2%) | 730 (26.0%) | 705 (25.9%) | 715 (25.5%) | <0.001 |
| High-Mid | 348 (25.6%) | 406 (27.8%) | 263 (23.8%) | 259 (22.3%) | 322 (24.4%) | 320 (23.5%) | 452 (28.5%) | 495 (20.5%) | 675 (24.1%) | 650 (23.9%) | 590 (21.1%) | <0.001 |
| High-Mid | 372 (27.4%) | 346 (23.7%) | 270 (24.5%) | 332 (28.5%) | 392 (19.7%) | 292 (21.5%) | 366 (23.0%) | 600 (24.8%) | 665 (23.7%) | 640 (23.5%) | 735 (26.3%) | <0.001 |
| **Insurance** |  |  |  |  |  |  |  |  |  |  |  |  |
| Medicare | 961 (69.8%) | 1059 (70.3%) | 833 (72.7%) | 862 (71.8%) | 987 (73.5%) | 917 (66.0%) | 1090 (67.5%) | 1795 (72.2%) | 2035 (71.2%) | 1830 (66.3%) | 1960 (68.5%) | <0.001 |
| Medicaid | 99 (7.2%) | 68 (4.5%) | 36 (3.1%) | 67 (5.6%) | 73 (5.4%) | 175 (12.6%) | 140 (8.7%) | 160 (6.4%) | 215 (7.5%) | 220 (8.0%) | 255 (8.9%) | <0.001 |
| Private | 279 (20.3%) | 328 (21.8%) | 221 (19.3%) | 232 (19.3%) | 218 (16.2%) | 246 (17.7%) | 321 (19.9%) | 425 (17.1%) | 480 (16.8%) | 565 (20.5%) | 480 (16.8%) | <0.001 |
| Self-Pay | 34 (2.5%) | 32 (2.1%) | 41 (3.6%) | 24 (2.0%) | 44 (3.3%) | 32 (2.3%) | 51 (3.2%) | 55 (2.2%) | 80 (2.8%) | 85 (3.1%) | 110 (3.8%) | <0.001 |
| **Comorbidities** |  |  |  |  |  |  |  |  |  |  |  |  |
| Obesity | 104 (7.6%) | 73 (4.8%) | 116 (10.1%) | 112 (9.3%) | 110 (8.2%) | 168 (12.1%) | 263 (16.3%) | 340 (13.7%) | 415 (14.5%) | 500 (18.1%) | 630 (22.0%) | <0.001 |
| Hypertension | 649 (47.1%) | 649 (43.0%) | 588 (51.3%) | 628 (52.3%) | 798 (59.5%) | 763 (54.9%) | 1003 (62.1%) | 1470 (59.2%) | 1745 (61.0%) | 1755 (63.5%) | 1960 (68.4%) | 0.158 |
| Smoking | 230 (16.7%) | 160 (10.6%) | 197 (17.2%) | 185 (15.4%) | 242 (18.1%) | 283 (20.4%) | 447 (27.7%) | 610 (24.5%) | 800 (2.0%) | 895 (32.4%) | 1010 (35.3%) | <0.001 |
| Dyslipidemia | 292 (21.2%) | 344 (22.8%) | 237 (20.7%) | 372 (31.0%) | 435 (32.5%) | 423 (30.4%) | 612 (37.9%) | 860 (34.6%) | 1240 (43.4%) | 1230 (44.5%) | 1315 (45.9%) | <0.001 |
| **Past Medical History** |  |  |  |  |  |  |  |  |  |  |  |  |
| PVD | 65 (4.6%) | 111 (7.4%) | 75 (6.5%) | 101 (8.4%) | 63 (4.7%) | 121 (8.7%) | 114 (7.1%) | 195 (7.8%) | 260 (9.1%) | 280 (10.1%) | 245 (8.6%) | <0.001 |
| CRF | 137 (9.9%) | 256 (17.0%) | 229 (20.0%) | 270 (22.5%) | 300 (22.4%) | 336 (24.2%) | 461 (28.5%) | 800 (32.2%) | 915 (32.0%) | 875 (31.6%) | 975 (34.0%) | <0.001 |
| CKD | 363 (26.4%) | 366 (24.2%) | 337 (29.4%) | 251 (20.9%) | 427 (31.9%) | 408 (29.4%) | 496 (30.7%) | 875 (35.2%) | 1030 (36.0%) | 880 (31.8%) | 810 (28.3%) | 0.38 |
| **Elixhauser score**  Mean (SD) | 3.9209 (6.53) | 4.2614 (7.05) | 5.1454 (7.34) | 4.7673 (7.21) | 5.6364 (8.17) | 5.2847 (7.75) | 6.1408 (8.57) | 5.8438 (8.53) | 6.0704 (9.18) | 5.7883 (9.04) | 5.9463 (8.64) | 0.014 |
| **Outcomes** |  |  |  |  |  |  |  |  |  |  |  |  |
| Cardiogenic shock | <11*  (0.7%) | <11*  (0.3%) | <11*  (0.8%) | 15  (1.2%) | <11*  (0.4%) | 20  (1.4%) | 38  (2.4%) | 75  (3.0%) | 100 (3.5%) | 90  (3.3%) | 75  (2.6%) | <0.001 |
| Ventricular tachycardia | 57 (4.1%) | 104 (6.9%) | 59 (5.1%) | 106 (8.8%) | 101 (7.5%) | 101 (7.3%) | 127 (7.9%) | 215 (8.7%) | 265 (9.3%) | 265 (9.6%) | 345 (12.0%) | <0.001 |
| Atrial fibrillation | 638 (46.3%) | 725 (48.0%) | 564 (49.2%) | 480 (40.0%) | 580 (43.3%) | 616 (44.3%) | 769 (47.6%) | 1300 (52.3%) | 1565 (54.7%) | 1360 (49.2%) | 1605 (56.0%) | <0.001 |

*CAD= coronary artery disease. CKD=chronic kidney disease. PVD= Peripheral vascular disease. *Per the requirements of the HCUP, cells less or equal to 10 are noted as < 11*

**Supplementary Table 3:** Predictors of in-hospital mortality among diabetes patients with HF and HCM

|  |  | |
| --- | --- | --- |
|  | **OR (95% CI)** | **P value** |
| **Age** |  |  |
| <55 | Ref | Ref |
| 55 - 64 | 3.87 (1.45-10.34) | 0.007 |
| 65 - 74 | 7.58 (3.03-18.99) | <0.001 |
| 75 - 84 | 9.14 (3.68-22.70) | <0.001 |
| >85 | 7.49 (2.88-19.45) | <0.001 |
| Mean (SD) | 1.04 (1.03-1.05) | <0.001 |
| **Gender** |  |  |
| Male | Ref | Ref |
| Female | 1.50 (1.08-2.08) | 0.016 |
| **Race** |  |  |
| White | Ref | Ref |
| Black | 0.78 (0.54-1.13) | 0.191 |
| Hispanic | 0.35 (0.14-0.86) | 0.023 |
| Asian | 1.20 (0.89-0.40) | 0.995 |
| **Income** |  |  |
| Low | Ref | Ref |
| Low-Mid | 1.84 (1.23-2.75) | 0.003 |
| High-Mid | 1.62 (1.06-2.50) | 0.028 |
| High | 1.09 (0.68-1.76) | 0.719 |
| **Insurance** |  |  |
| Medicare | Ref | Ref |
| Medicaid | 0.21 (0.09-0.52) | <0.001 |
| Private Insurance | 0.41 (0.24-0.76) | 0.001 |
| Self-Pay | 2.80 (0.83-2.10) | 0.995 |
| **Comorbidities** |  |  |
| Obesity | 0.83 (0.59-1.17) | 0.288 |
| Hypertension | 0.86 (0.63-1.18) | 0.355 |
| Smoking | 0.29 (0.17-0.49) | <0.001 |
| Dyslipidemia | 0.83 (0.61-1.11) | 0.204 |
| **Past Medical History** |  |  |
| PVD | 2.98 (2.11-4.21) | <0.001 |
| CKD | 1.35 (1.004-1.82) | 0.047 |
| CAD | 1.54 (1.14-2.07) | 0.004 |
| **Elixhauser score** | 1.09 (1.07-1.11) | <0.001 |

*CAD= coronary artery disease. CKD= chronic kidney disease. PVD= Peripheral vascular disease.*

**Supplementary Table 4:** Predictors of cardiogenic shock among diabetes patients with HF and HCM

|  | **OR (95% CI)** | **P value** |
| --- | --- | --- |
| **Age** |  |  |
| <55 | Ref | Ref |
| 55 - 64 | 0.93 (0.49-1.79) | 0.832 |
| 65 - 74 | 0.94 (0.49-1.81) | 0.857 |
| 75 - 84 | 0.71 (0.32-1.57) | 0.399 |
| >85 | 1.26 (0.53-3.04) | 0.6 |
| Mean | 1.14 (0.93-1.65) | 0.845 |
| **Gender** |  |  |
| Male | Ref | Ref |
| Female | 0.47 (0.31-0.72) | <0.001 |
| **Race** |  |  |
| White | Ref | Ref |
| Black | 0.54 (0.30-1.00) | 0.051 |
| Hispanic | 1.53 (0.82-1.42) | 0.991 |
| Asian | 4.88 (2.14-11.13) | <0.001 |
| **Income** |  |  |
| Low | Ref | Ref |
| Low-Mid | 0.08 (0.02-0.30) | <0.001 |
| High-Mid | 2.99 (1.73-5.17) | <0.001 |
| High | 0.80 (0.42-1.54) | 0.506 |
| **Insurance** |  |  |
| Medicare | Ref | Ref |
| Medicaid | 2.41 (1.13-5.15) | 0.023 |
| Private Insurance | 4.00 (2.32-6.89) | <0.001 |
| Self-Pay | 1.18 (0.94-3.01) | 0.996 |
| **Comorbidities** |  |  |
| Obesity | 2.27 (1.42-3.65) | <0.001 |
| Hypertension | 0.65 (0.43-1.996) | 0.408 |
| Smoking | 0.92 (0.71-1.49) | 0.21 |
| Dyslipidemia | 1.60 (0.94-1.91) | 0.071 |
| **Past Medical History** |  |  |
| PVD | 1.50 (0.85-2.65) | 0.163 |
| CKD | 1.31 (1.19-1.50) | <0.001 |
| CAD | 2.41 (1.57-3.70) | <0.001 |
| **Elixhauser score** | 1.11 (1.08-1.13) | <0.001 |

*CAD= coronary artery disease. CKD= chronic kidney disease. PVD= Peripheral vascular disease.*

**Supplementary Table 5:** Predictors of atrial fibrillation among diabetes patients with HF and HCM

|  |  | |
| --- | --- | --- |
|  | **OR (95% CI)** | **P value** |
| **Age** |  |  |
| <55 | Ref | Ref |
| 55 - 64 | 1.66 (1.51-1.82) | <0.001 |
| 65 - 74 | 1.74 (1.57-1.93) | <0.001 |
| 75 - 84 | 2.25 (2.03-2.49) | <0.001 |
| >85 | 1.95 (1.75-2.18) | <0.001 |
| Mean | 1.84 (1.64-2.11) | <0.001 |
| **Gender** |  |  |
| Male | Ref | Ref |
| Female | 0.67 (0.63-0.71) | <0.001 |
| **Race** |  |  |
| White | Ref | Ref |
| Black | 0.52 (0.48-0.56) | <0.001 |
| Hispanic | 0.70 (0.62-0.79) | <0.001 |
| Asian | 0.89 (0.74-1.08) | 0.233 |
| **Income** |  |  |
| Low | Ref | Ref |
| Low-Mid | 0.98 (0.92-1.06) | 0.671 |
| High-Mid | 1.05 (0.97-1.13) | 0.231 |
| High | 1.09 (1.01-1.18) | 0.026 |
| **Insurance** |  |  |
| Medicare | Ref | Ref |
| Medicaid | 0.69 (0.61-0.78) | <0.001 |
| Private Insurance | 0.85 (0.78-0.92) | <0.001 |
| Self-Pay | 0.42 (0.34-0.51) | <0.001 |
| **Comorbidities** |  |  |
| Obesity | 1.42 (1.12-1.95) | <0.001 |
| Hypertension | 0.95 (0.89-1.98) | 0.111 |
| Smoking | 1.36 (0.89-1.98) | 0.224 |
| Dyslipidemia | 1.02 (0.97-1.08) | 0.422 |
| **Past Medical History** |  |  |
| PVD | 1.87 (1.79-1.95) | 0.002 |
| CKD | 0.998 (0.94-1.06) | 0.941 |
| CAD | 1.08 (1.02-1.14) | 0.008 |
| **Elixhauser score** | 1.01 (1.01-1.02) | <0.001 |

*CAD= coronary artery disease. CKD= chronic kidney disease. PVD= Peripheral vascular disease.*

**Supplementary Table 5:** Predictors of ventricular fibrillation among diabetes patients with HF and HCM

|  |  | |
| --- | --- | --- |
|  | **OR (95% CI)** | **P value** |
| **Age** |  |  |
| <55 | Ref | Ref |
| 55 - 64 | 1.79 (1.33-2.40) | <0.001 |
| 65 - 74 | 1.57 (1.14-2.16) | 0.006 |
| 75 - 84 | 0.97 (0.68-1.40) | 0.888 |
| >85 | 1.14 (0.76-1.72) | 0.534 |
| Mean | 1.12 (1.01-1.25) | 0.023 |
| **Gender** |  |  |
| Male | Ref | Ref |
| Female | 0.63 (0.53-0.77) | <0.001 |
| **Race** |  |  |
| White | Ref | Ref |
| Black | 1.22 (0.98-1.52) | 0.071 |
| Hispanic | 1.12 (0.78-1.61) | 0.527 |
| Asian | 1.25 (0.71-2.22) | 0.439 |
| Native American | 2.21 (0.84-5.77) | 0.106 |
| Other | 0.59 (0.31-1.12) | 0.107 |
| **Income** |  |  |
| Low | Ref | Ref |
| Low-Mid | 1.33 (1.04-1.69) | 0.022 |
| High-Mid | 1.02 (0.77-1.34) | 0.898 |
| High | 1.38 (1.06-1.80) | 0.017 |
| **Insurance** |  |  |
| Medicare | Ref | Ref |
| Medicaid | 0.83 (0.57-1.21) | 0.333 |
| Private Insurance | 1.31 (1.01-1.70) | 0.04 |
| Self-Pay | 0.97 (0.54-1.75) | 0.924 |
| No Charge | 2.50 (0.92-6.81) | 0.073 |
| Other | 2.87 (1.55-5.29) | <0.001 |
| **Comorbidities** |  |  |
| Obesity | 1.12 (0.90-1.39) | 0.297 |
| Hypertension | 0.84 (0.70-1.02) | 0.075 |
| Smoking | 0.94 (0.62-1.92) | 0.21 |
| Dyslipidemia | 1.02 (0.85-1.23) | 0.812 |
| **Past Medical History** |  |  |
| PVD | 1.62 (1.46-1.87) | 0.006 |
| Renal Failure | 1.69 (1.56-1.85) | <0.001 |
| CAD | 0.99 (0.82-1.20) | 0.953 |
| Elixhauser score | 1.03 (1.02-1.04) | <0.001 |
